# The Influence of Increased Dairy Product Consumption, as Part of a Lifestyle Modification Intervention, on Diet Quality and Eating Patterns in Female Adolescents with Overweight/Obesity

**DOI:** 10.3390/children9111703

**Published:** 2022-11-06

**Authors:** Emily C. Fraschetti, Lauren E. Skelly, Mavra Ahmed, Emma C. Biancaniello, Panagiota Klentrou, Andrea R. Josse

**Affiliations:** 1 School of Kinesiology and Health Science, Faculty of Health, York University, Toronto, ON M3J 1P3, Canada; 2 Department of Nutritional Sciences, Temerty Faculty of Medicine, University of Toronto, Toronto, ON M5S 1A8, Canada; 3 Joannah and Brian Lawson Centre for Child Nutrition, Temerty Faculty of Medicine, University of Toronto, Toronto, ON M5S 1A8, Canada; 4 School of Food and Nutritional Sciences, Brescia University College, London, ON N6G 1H2, Canada; 5 Department of Kinesiology, Faculty of Applied Health Sciences, Brock University, St. Catharines, ON L2S 3A1, Canada

**Keywords:** eating patterns, snacking, eating behaviour, dairy products, adolescents, obesity, intervention study, weight management

## Abstract

Our study examined how increased dairy consumption versus habitually low dairy consumption, against a background of healthy eating (and exercise), influenced diet quality, nutrient intake, and snacking in Canadian female adolescents (14.8 ± 2.2 years) with overweight/obesity (OW/OB). We also explored dairy consumption patterns in the group consuming dairy products. Participants were randomized into two groups: higher/recommended dairy (RDa; 4 svg/d; *n* = 24) or low dairy (LDa; 0–2 svg/d; *n* = 23). Both groups participated in a 12-week, eucaloric, lifestyle modification intervention consisting of exercise training and nutritional counseling. The intervention increased the total Canadian Healthy Eating Index score (*p* < 0.001) with no differences between groups. The “other food” sub-score improved more in RDa than LDa (*p* = 0.02), and the “saturated fat” sub-score increased more in LDa than RDa (*p* = 0.02). The intervention significantly increased the consumption of dairy-related nutrients more in RDa than LDa (*p* < 0.05). The intervention also decreased snack size in both groups (*p* = 0.01) and improved percentage of healthy snack energy intake more in RDa than LDa (*p* = 0.04). More servings of dairy products were consumed as snacks than at breakfast, lunch, or dinner (*p* < 0.05). Thus, our study improved diet quality, and dairy product consumption improved intakes of key related nutrients and snack consumption in adolescents with OW/OB.

## 1. Introduction

Within Canada, there is a high prevalence (~32%) of overweight and obesity (OW/OB) among females aged 12–17 years old [1]. This high prevalence of OW/OB in female youth is concerning, in part, because most youth with OW/OB become adults with OW/OB [2] and are at increased risk for chronic diseases [3]. Lifestyle modification, including diet and exercise programs, are a safe, effective, and accessible way to reduce OW/OB in adolescents [4]. Further, the consumption of dairy products as part of a healthy diet is associated with improved diet quality, higher intakes of key shortfall nutrients such as calcium and vitamin D [5,6,7], and reduced risk of OW/OB among children and adolescents [8]. Despite these favourable associations, adolescents are under-consuming dairy products, with only 26% of Canadian adolescents aged 13–18 years old meeting recommendations for the “milk and alternatives” food group in 2015 [9] and with female adolescents generally consuming fewer servings of dairy products than males [10,11]. This may have a negative impact on overall nutrient intakes of particular importance to growth and development, as “milk and alternatives” foods were reported to provide over 50% of the daily calcium intake, 45% of vitamin D, and 40% of vitamin B12 in this population in 2015 [9]. In addition, lower diet quality has been reported in adolescents with OW/OB compared to adolescents with normal weight [12]. Thus, increasing dairy product consumption, alongside the provision of general healthy eating advice, may be one strategy to improve eating patterns in adolescents with OW/OB and may enable them to better achieve the current nutrient intake recommendations.

Snacking, typically defined as eating occurrences between meals [13], is a common practice among adolescents. Indeed, snacks have been shown to contribute to approximately 25% of daily energy intake (DEI) in Canadian adolescents, and 85% of Canadian adolescents report consuming at least one snack per day [14]. Thus, the consumption of nutrient-dense and satiating snacks may provide a significant opportunity for adolescents to reach their daily nutrient recommendations and may also benefit weight management efforts [15,16]. Dairy products (predominantly milk, yogurt, and cheese) are nutrient-dense foods sometimes consumed as snacks. In Canada, 23% of snacks consumed by adolescents were from the “milk and alternatives” food group [17], and snacks represent the second most common eating occasion for dairy products, after breakfast [14]. Given the favourable nutrient profile of dairy products [18], increasing the consumption of dairy products as snacks may be a good dietary strategy for adolescents with OW/OB to improve snacking quality (i.e., to replace less healthful snacks) and nutrient intake. However, limited research exists aiming to characterize features of snacking during a period of increased dairy product intake in conjunction with general healthy eating advice in adolescents with OW/OB. There is also limited research in this demographic examining how increased intakes of dairy products impact diet quality scores, which are useful assessments in interventions involving diet counselling targeting multiple food behaviours [19]. Moreover, research into how adolescents with OW/OB choose to autonomously consume different dairy products from a dietary intake and eating pattern perspective (i.e., at which eating occasion do they consume dairy products) is warranted to help best tailor future dietary interventions and personalized nutrition strategies for this demographic.

Our group recently conducted a randomized controlled trial (RCT) where female adolescents with OW/OB participated in a 12-week lifestyle modification, weight management intervention consisting of exercise training, and nutritional counselling with either low dairy product intake or higher dairy product intake. The main outcome of our trial was body composition [20]. The aim of the present secondary investigation was to examine how increased dairy product consumption versus habitually low dairy product consumption, against a background of healthy eating advice (and exercise), influenced diet quality, nutrient intake, and indices of snacking in these participants. A secondary objective was to specifically explore dairy product eating patterns in the group consuming dairy products. We hypothesized that increased dairy product consumption would improve diet quality, nutrient intake, and healthy snack consumption to a greater extent than a low-dairy-product diet and that dairy products would be most often consumed during breakfast and snack occasions.

## 2. Materials and Methods

### 2.1. Participants

Participants were recruited using social media, posters, flyers, and information sheets posted at Brock University (St. Catharines, ON, Canada), within the community (community centres, libraries, family health clinics/doctors’ office), and schools (elementary and high schools in the Niagara region). Participant descriptive data are shown in Table 1. The RCT took place at Brock University from June 2016 to October 2018. Information regarding recruitment, written informed consent and assent, and the inclusion/exclusion criteria have been described elsewhere [20]. The study was approved by Brock University’s Biosciences Research Ethics Board (REB 14-284, 7/17/2015), and the main trial was registered at clinicaltrials.gov (NCT#02581813).

### 2.2. Study Design

The present study is a secondary analysis of diet quality, nutrient intake, and snacking patterns from the “I.D.E.A.L (Improving Diet, Exercise and Lifestyle) for Adolescents” study, a 12-week randomized, controlled, parallel intervention study primarily designed to assess the effect of consuming the recommended four servings per day (svg/d) of dairy products vs. a low dairy product diet (0–2 svg/d), along with mixed-mode exercise training as part of a weight management intervention, on body composition in female adolescents (aged 10–18 years) with OW/OB [20]. As previously described [20], participants were stratified by body mass index (BMI) percentile (overweight or obese) and randomly assigned (using a random number generator) to one of two intervention groups (recommended dairy product intake (RDa) and low dairy product intake (LDa)) and a no-intervention control group using an unblocked, random allocation ratio of 2:2:1 (CONSORT diagram and checklist, Supplementary Figure S1 and Table S1, respectively). Participants from the intervention groups only were included in the present secondary analysis. Two independent study coordinators enrolled the participants and assigned them to the groups. Further details regarding randomization in accordance with CONSORT are reported in the main trial [20]. Both intervention groups (RDa and LDa) were provided with detailed, individualized dietary advice from a registered dietitian (RD); the same RD was employed throughout the study for both groups. RDa was also provided with 4 svg/d of dairy products, while LDa was instructed to maintain their habitual low dairy product intake of ≤2 svg/d. Prior to the start of the intervention, participants visited the laboratory for baseline testing and were instructed on how to complete a food record. Participants also took part in a supervised individualized exercise program for the 12 weeks that followed the same principles for both groups.

### 2.3. Anthropometrics and Body Composition

At weeks 0 and 12, body mass was measured with a standard scale (Digital Physician Scale, Rice Lake Weighing Systems, Rice Lake, WI) to the nearest 0.1 kg, wearing light clothes and no shoes. Height was measured using a stadiometer (Seca 213 Portable Stadiometer, CME Corp., Warwick, RI, USA) to nearest 0.1 cm with no shoes. BMI was calculated. Waist circumference, body composition (fat mass, lean mass and % body fat; assessed *via* ultrasound using the BodyMetrix (BMX BodyMetrix System, BX-2000, IntelaMetrix, Inc., Livermore, CA, USA)), and somatic maturity were also assessed and reported elsewhere [20].

### 2.4. Exercise Intervention

Both intervention groups completed an individualized structured exercise program over the 12 weeks (3 times/week). A detailed description of the exercise intervention has been published elsewhere [20]. Briefly, the exercise sessions each lasted 60–90 min and included a warm-up, followed by 20+ min of aerobic exercise and 15–20 min of resistance or plyometric exercise accompanied by a cool-down. Following the structured exercise, participants in the RDa group consumed 1 cup (250 mL) of 1% chocolate milk, and participants in the LDa group consumed 1 cup of a carbohydrate-based electrolyte drink. The post-exercise chocolate milk intake (1 cup, 3 times/week) represented the only time the RDa group was explicitly instructed to consume a dairy product during the intervention and was done in accordance with best practices [21] to provide nutrients post exercise to facilitate recovery and to support our primary endpoint of body composition change, as done in [22,23].

### 2.5. Dietary Intervention

A RD provided individual 1-hour dietary counselling sessions to participants in both groups at five timepoints during the intervention (weeks 0, 2, 4, 8, 12). Participants’ parent(s)/guardian(s) also attended the dietary counselling sessions. Both groups were instructed to maintain their energy intake throughout the weight management intervention. Energy requirements were calculated for each participant using predictive equations from the Institutes of Medicine for girls with OW with a sedentary activity factor [24,25]. All intervention participants were counseled to consume a healthy, nutrient-dense diet in accordance with the appropriate number of servings in the 2007 Canada’s Food Guide (CFG) [26] for their age (except for the dairy food group). Participants were counselled to avoid/minimize processed foods (pre-packaged, preserved, and “convenience” foods), foods high in “bad” (primarily trans) fats, sugar-sweetened beverages (SSBs), pastries, and confectionary and to replace these foods with more nutrient-dense options, such as fruits and vegetables, high-fibre foods, whole grains, lean meats, and meat alternatives. Participants were also provided with guidelines regarding portion sizes of common foods, which were reviewed with participants using food models and measuring tools (i.e., measuring cups). In addition, through dietary advice from the RD, participants were asked to maintain a total energy macronutrient distribution of 55%, 20%, and 25% for carbohydrate, protein, and fat, respectively. These relative macronutrient intakes are within the acceptable macronutrient distribution ranges, and the higher protein intakes were to support our primary endpoint of body composition change, as done in [22,23]. As mentioned, the RDa group was instructed to consume 4 svg/d of mixed dairy products, as per the recommendations at the time for their age from the 2007 CFG [26]. Dairy products were provided to the participants every 2 weeks. Per day, they were instructed to consume 2 cups of 1% milk (white and chocolate), 2 × 100 g cartons of 0% or 2% milk fat Greek yogurt (of their preferred flavour), and 42 g of full-fat cheddar or marble cheese. The LDa group was instructed to maintain their habitually low dairy diet (0–2 svg/d of dairy products) and to avoid consuming calcium-fortified beverages and juices.

Both groups were encouraged by the RD to consume snacks throughout the day. Importantly, there were no concrete recommendations for how frequently they should snack each day or how much they should eat at each snack. Both groups were encouraged to include a source of protein and a fruit or vegetable or whole grain with each snack to help promote protein intake and satiety. In addition, and commensurate with the general dietary advice provided to all participants, the RD discouraged the consumption of snacks high in sodium, sugars (added), and fat in favour of more nutrient-dense options. Participants were also counselled to “snack mindfully”, meaning they were guided to try to avoid snacking out of boredom or when distracted (e.g., while watching television). Participants were discouraged from “mindlessly” consuming food after dinner unless they were hungry. If they were hungry, they were encouraged to consume a healthy snack based on the same aforementioned principles. For participants in the RDa group, the RD suggested that eating dairy products at meals and incorporating them into snacks throughout the day may help the participants reach their required daily dairy servings (4 svg/d), but they were not explicitly told to consume their dairy products as snacks and could freely incorporate them into any eating occasion (apart from the instruction to consume one cup of chocolate milk following each exercise session; 3 times/week).

### 2.6. Food Record Analysis

Seven-day food records were completed at weeks 0 and 12. Food items were weighed or measured using household measures (i.e., measuring cups and spoons) and reviewed by the RD. Data from these records were used in the present analysis. Three-day food records were also completed at weeks 2, 4, and 8 to assess dietary intake, track compliance, and to help the RD provide targeted guidance to participants (and their parent(s)/guardian(s)) during the counselling sessions. Following completion of the entire study, two other RDs who were not involved in the dietary counselling during the intervention analyzed the food records using the ESHA dietary analysis program (Food Processor SQL Version 10.14.2, ESHA Research, Salem, OR, USA) to assess the dietary endpoints herein (Canadian Healthy Eating Index (HEI-C), nutrient intakes, snacking, and dairy product eating patterns (in RDa only)).

Seven-day food records were utilized for the snacking and dairy product eating pattern analyses. For the HEI-C and nutrient intake analyses, three days were selected from the week 0 and 12 records (the first weekend day and first two weekdays). For each participant, mean 7-day and 3-day energy intakes reported at weeks 0 and 12 were compared with energy requirements calculated for each participant. To account for under- and over-reporting, a ratio of energy intake to energy requirement was calculated. Under- and over-reporters, defined as those having a ratio of <0.50 and >1.98 [27], respectively, were removed from the respective analyses, which slightly reduced the sample size for the diet quality and nutrient intake analyses to LDa *n* = 20 and RDa *n* = 23 and the snacking analysis to LDa *n* = 21 and RDa *n* = 22.

### 2.7. Canadian Healthy Eating Index (HEI-C) and Nutrient Intake Analyses

To assess diet quality, HEI-C scores [28] were determined from 3 representative days of the 7-day food records collected pre- and post intervention using the 2007 CFG age- and sex-specific recommended serving sizes [26]. The HEI-C score was calculated out of 100 points and was obtained by adding up sub-scores for adequacy and moderation components, with higher total scores reflecting higher quality diets. There are nine adequacy components (total vegetables and fruit, whole fruit, dark green and orange vegetables, total grain products, whole grains, milk and alternatives, meat and alternatives, and unsaturated fats) and three moderation components (saturated fats, sodium, and other food). RDs categorized each food item consumed on each day into the different categories and determined serving sizes. Multicomponent foods were disaggregated into individual ingredients, and the top three ingredients based on weight were determined and included in the HEI-C score (e.g., a burger contributed towards the meat and alternatives (beef), total grain (bun), and total vegetables and fruit (tomato) components).

To assess the nutrient intakes of our study participants and how they changed during the intervention, intakes of several macronutrients and micronutrients were assessed from the same 3 days selected for the HEI-C analysis. These were compared to the corresponding estimated average requirements (EAR), recommended dietary allowances (RDA), or adequate intakes (AI). Of note, this study was not primarily designed to assess habitual nutrient intakes at a population level, but comparing our intake data to the general nutrient requirements and recommendations (i.e., DRIs) provides insight on the efficacy of our dietary intervention in our study sample.

### 2.8. Snacking Characteristics and Eating Patterns Analyses

For these analyses, all 7 days of each food record were assessed, and all eating occasions (i.e., snacks and meals) were identified and classified. We defined a snack as “any food/drink consumed between meals” [13,15]. In most instances (~51% of food records), eating occasions were labeled on the food record, as participants were instructed to do so by the RD (i.e., to designate breakfast, morning snack, snack 1, snack 2, lunch, etc.). When the eating occasion was not explicitly specified (~46% of food records), timing of intakes was usually provided (i.e., 10:00 a.m., 4:30 p.m., etc.), and different/discrete eating occasions were assumed when foods were consumed at least 15 min apart. For these records, we took the approach of identifying meals first based on reasonable timing and energy intakes and then identified the remainder as snacks (between meals). Beverages containing calories/nutrients (e.g., coffee-based beverage, smoothie) consumed in the absence of food and not as part of a meal were designated as snacks, as previously done in [29]. For a few instances, where eating occasion and/or timing was not clear, we took a conservative approach and designated them as meals instead of snacks. Lastly, there were some food records (~3% of food records) that did not follow any of the aforementioned patterns. For these records, the RDs determined the eating occasion designations. Given that the participants in the LDa and RDa groups were explicitly asked to consume a carbohydrate-based electrolyte drink or chocolate milk following exercise, respectively, these items were not included as a snack for the analyses assessing snacking or dairy product eating patterns (discussed below), as we wanted our analyses to best reflect participants’ volitional choices.

Once snacks were identified, they were placed into one of eleven categories, as seen in Table 2. Details on the 3–5 most popular foods within each category are also presented. “Healthy” snacks included those primarily from the 2007 CFG food groups (categories 1–4), and “unhealthy” snacks consisted of the other seven categories (categories 5–11).

Snacking characteristics and relative consumption of snacks from the different categories were assessed at weeks 0 and 12. Characteristics included (a) absolute snack energy intake (kcal consumed as snacks/d), (b) relative snack energy intake (percent of total kcal/d consumed as snacks), (c) snacking frequency (number of snacks/d), and (d) energy intake per snack (kcal/snack occasion). The total daily energy intake (kcals) of each snack category was expressed relative to the total daily snack energy intake (kcals). Individual days where participants did not consume any snacks were not included in the snack category analysis. Energy from amalgamated categories of “healthy” and “unhealthy” snacks were also expressed as a proportion of total snack energy. Given that we conducted our analyses relative to the energy (kcal) content of snacks, zero-calorie beverages such as water, plain coffee/tea, and diet soda were not included as snacks in this analysis (21 cases across all food records). Homemade mixed snacks, such as sandwiches and smoothies, were disaggregated into their main ingredient constituents and placed into separate categories, as previously done in [30]. Mixed snacks purchased outside the home, such as sandwiches, pizza, and smoothies (i.e., from a fast-food chain), were not disaggregated and were placed into the “processed/mixed foods” or “drinks” categories.

### 2.9. Dairy Product Eating Patterns Analysis

We determined dairy product eating patterns in the RDa group at week 12 by comparing the number of servings of each dairy product consumed at breakfast, lunch, dinner, and snack. This analysis focused specifically on milk, cheese, and yogurt, as this reflected the products provided to the participants during the intervention. We also assessed “total dairy” as the sum of these dairy products, and “other dairy”, which included additional dairy products not included in 2007 CFG (i.e., cream, sour cream, cream cheese, frozen yogurt, and ice cream) at the different eating occasions. Products in the “total dairy” category were quantified as svg/d, and products in the “other dairy” category were quantified based on energy (kcals) per day and expressed as a proportion of DEI similar to how “other food” in CFG are quantified in diet quality analyses [28].

### 2.10. Statistical Analysis

For the HEI-C, nutrient intake and snacking characteristics analyses, two-way repeated measures analyses of variance (RM-ANOVA) were conducted, with group (LDa, RDa) as the between factor and time (weeks 0, 12) as the within factor. Data were assessed for normality by analyzing skewness and kurtosis. Variables that were not normally distributed were log- or square-root-transformed. If the data remained not normally distributed following transformations, outliers (>±2 standard deviations [SD]) were identified in the non-transformed data set and replaced with the corresponding 2 SD limits. If normality improved, parametric tests were used. If not, non-parametric tests were used. The Wilcoxon signed-rank test to compare values at weeks 0 and 12 and the Mann–Whitney U test to compare changes over the intervention between groups were used for the Meat and Alternatives HEI-C sub-score and the snacking category data due to non-normal distributions. For the dairy product eating patterns analyses at week 12, servings of total dairy and types of dairy products were analyzed using a one-way RM-ANOVA to determine differences between eating occasions (breakfast, lunch, dinner, snack). When appropriate (i.e., following significant interactions in the two-way RM-ANOVA or a significant F value in the one-way RM-ANOVA), pairwise comparisons were performed using *t*-tests (two-way RM-ANOVA) or Fisher’s least significance difference tests (one-way RM-ANOVA). Significance was set at *p* ≤ 0.05. Statistical analyses were performed using SPSS version 27.0 (SPSS, Chicago, IL, USA) and GraphPad Prism 9.0.2 (GraphPad Software, La Jolla, CA, USA). Results are presented as mean (SD).

## 3. Results

### 3.1. Diet Quality Analyses

#### 3.1.1. Canadian Healthy Eating Index (HEI-C; Table 3)

The 12-week intervention increased the total HEI-C score, with no differences between groups. There were significant interactions for the “milk and alternatives” and the “other food” sub-scores, where RDa improved more than LDa for both. In contrast, the “saturated fats” sub-score improved in LDa more than RDa. In both groups, there were improvements in the “total vegetables and fruit”, “dark green and orange vegetables”, “whole grain”, and “meat and alternatives” sub-scores, with no differences between groups. There was a decrease in the “total grains” sub-score in both groups and no changes over time or between groups for the “whole fruits”, “unsaturated fats”, or “sodium” sub-scores.

#### 3.1.2. Nutrient Intakes (Table 4)

Total protein, riboflavin, vitamin B12, vitamin D, calcium, phosphorus, and potassium intakes increased to a greater extent in RDa compared to LDa over the intervention. Iron intake decreased to a greater extent in RDa compared to LDa, and sugar intake decreased to a greater extent in LDa compared to RDa over the intervention. Alpha-linolenic acid, niacin, magnesium, and zinc improved in both groups. At week 12, the proportion of RDa and LDa participants with a mean intake below the EAR was 13% and 100%, respectively, for calcium; 4% and 45%, respectively, for vitamin B12; and 0% and 90%, respectively, for phosphorus. Relatedly, the proportion of RDa and LDa participants with a mean intake below the RDA was 39% and 100%, respectively, for calcium; 4% and 50%, respectively, for vitamin B12; and 22% and 90%, respectively, for phosphorus.

**Table 3:** Canadian Healthy Eating Index (HEI-C) scores in adolescent females with overweight/obesity in the low dairy product intake (LDa) and recommended dairy product intake (RDa) groups at weeks 0 and 12 of the intervention.

	LDa (*n* = 20)			RDa (*n* = 23)			*p*-Values		
HEI-C Score (Max)	Wk 0	Wk 12	Change	Wk 0	Wk 12	Change	G	T	Int
Total HEI-C (100)	51.4 (13.0)	65.8 (10.5)	14.4 (15.9)	50.5 (12.3)	70.9 (10.6)	20.4 (15.2)	0.44	**<0.001**	0.21
Total vegetables and fruit (10)	3.8 (2.4)	6.2 (2.9)	2.4 (3.2)	3.7 (2.5)	6.0 (2.9)	2.2 (3.3)	0.82	**<0.001**	0.84
Whole fruit (5)	2.9 (2.0)	3.6 (1.7)	0.7 (2.8)	2.9 (2.0)	3.7 (1.7)	0.8 (2.6)	0.94	0.071	0.90
Dark green and orange vegetables (5)	1.2 (1.3)	3.4 (1.9)	2.2 (2.0)	1.3 (1.2)	3.3 (1.6)	2.0 (1.9)	0.89	**<0.001**	0.68
Total grain products (5)	3.9 (1.2)	3.5 (0.9)	−0.4 (1.2)	4.1 (0.9)	3.1 (0.8)	−1.0 (1.0)	0.70	**<0.001**	0.060
Whole grains (5) ^‡^	0.8 (1.2)	2.6 (1.7)	1.8 (1.6)	1.4 (1.5)	2.0 (1.6)	0.6 (2.1)	0.83	**<0.001**	0.069
Milk and alternatives (10)	1.8 (1.5)	0.7 * (1.0)	−1.1 (1.6)	3.0 ^#^ (2.0)	9.0 *^#^ (1.2)	6.0 (2.5)	**<0.001**	**<0.001**	**<0.001**
Meat and alternatives (10)	7.9 (2.6)	9.4 (2.0)	1.5 (3.7)	7.5 (2.5)	8.5 (2.4)	1.0 (3.2)	--	**0.009**	0.68
Unsaturated fats (10) ^§^	8.5 (2.1)	9.3 (1.4)	0.8 (2.5)	8.5 (2.1)	8.8 (1.5)	0.3 (2.6)	0.50	0.17	0.52
Saturated fats (10) ^§^	5.6 (3.8)	8.0 * (1.9)	2.4 (3.4)	5.0 (2.8)	4.5 ^#^ (2.6)	−0.5 (4.1)	**0.003**	0.11	**0.016**
Sodium (10)	5.9 (3.4)	6.8 (2.8)	0.9 (4.8)	5.7 (2.6)	6.1 (3.1)	0.3 (3.5)	0.54	0.33	0.66
“Other food” (20)	9.2 (6.6)	12.5 (5.1)	3.3 (7.6)	7.3 (5.7)	16.0 *^#^ (4.9)	8.7 (7.0)	0.54	**<0.001**	**0.020**

Data are presented as mean (standard deviation). Significance set at *p* ≤ 0.05. Group (G), time (T), and interaction (Int) *p*-values are presented from the repeated-measures ANOVAs (*p* ≤ 0.05 presented in bold). The *p*-values from the Wilcoxon matched pairs signed-rank test to compare week 0 and 12 values and the Mann–Whitney U test to compare changes over the intervention between groups are presented for the meat and alternatives sub-score (no group *p*-value was obtained from this analysis, hence (--)). The symbols denote when analyses were run with square-root-transformed (‡) or outlier-treated (§) data sets and a significant difference over time within a group (*) or at a single timepoint between groups (#) based on paired-test tests or unpaired *t*-tests, respectively, conducted as post hoc testing following a significant interaction.

**Table 4:** Nutrient intake in adolescent females with overweight/obesity in the low dairy product intake (LDa) and recommended dairy product intake (RDa) groups at weeks 0 and 12 of the intervention.

	DRI		LDa (*n* = 20)			RDa (*n* = 23)			*p*-Values		
Nutrient			Wk0	Wk 12	Change	Wk0	Wk 12	Change	G	T	Int
Energy (kcal)	**-**		1637 (481)	1485 (204)	−151 (538)	1812 (431)	1749 (346)	−62 (430)	**0.019**	0.16	0.55
Protein (g/kg/d) †	0.76/0.71 ^a^	0.95/0.85 ^b^	0.91 (0.34)	0.97 (0.16)	0.06 (0.33)	0.86 (0.34)	1.17 *^#^ (0.30)	0.31 (0.24)	0.45	**<0.001**	**0.021**
Protein (g)	34/46 ^b^		68 (20)	73 (11)	5 (22)	66 (19)	91 *^#^ (16)	24 (18)	**0.053**	**<0.001**	**0.004**
Protein (% of energy)	10–30 ^d^		17 (4)	20 * (3)	3 (5)	15 ^#^ (3)	21 * (3)	6 (4)	0.49	**<0.001**	**0.023**
Fat (g)	**-**		64 (24)	60 (14)	−4 (27)	73 (17)	67 (19)	−6 (24)	0.073	0.20	0.83
Carbohydrate (g)	100 **^a^**	130 ^b^	204 (62)	172 (32)	−32 (76)	228 (65)	202 (52)	−26 (64)	**0.044**	**0.009**	0.81
Total fibre (g)	26 ^c^		15 (4)	19 (7)	4 (6)	14 (4)	15 (6)	0 (6)	0.14	**0.031**	0.057
Total sugar (g)	**-**		69 (36)	49 * (18)	−20 (44)	84 (34)	97 ^#^ (30)	13 (39)	**<0.001**	0.59	**0.011**
Total saturated fatty acids (g) †	As low as possible		21 (11)	16 (4)	−5 (10)	24 (8)	24 (7)	0 (8)	**0.001**	0.15	0.080
Alpha-linolenic acid (g) †	1.0/1.1 ^c^		1.0 (0.5)	1.4 (0.9)	0.3 (0.9)	1.1 (0.5)	1.4 (0.9)	0.4 (0.9)	0.46	**0.037**	0.59
Linoleic acid (g)	10/11 ^c^		8.3 (3.5)	10.1 (4.3)	1.9 (6.2)	8.6 (4.0)	8.8 (4.8)	0.3 (6.3)	0.55	0.27	0.41
Thiamin (mg) †	0.7/0.9 **^a^**	0.9/1.0 ^b^	1.0 (0.3)	1.2 (0.4)	0.2 (0.5)	1.3 (0.8)	1.1 (0.4)	−0.2 (0.6)	0.36	0.88	0.056
Riboflavin (mg) †	0.8/0.9 **^a^**	0.9/1.0 ^b^	1.0 (0.4)	1.1 (0.4)	0.0 (0.6)	1.3 (0.5)	1.8 *^#^ (0.3)	0.5 (0.5)	**<0.001**	**0.004**	**0.012**
Niacin (mg NE)	9/11 **^a^**	12/14 ^b^	25 (10)	30 (8)	5 (13)	22 (8)	26 (8)	4 (9)	0.10	**0.017**	0.84
Vitamin B6 (mg)	0.8/1.0 **^a^**	1.0/1.2 ^b^	1.1 (0.5)	1.3 (0.4)	0.2 (0.6)	1.1 (0.7)	1.2 (0.4)	0.1 (0.7)	0.74	0.11	0.51
Vitamin B12 (ug) †	1.5/2.0 **^a^**	1.8/2.4 ^b^	2.3 (1.5)	2.3 (1.3)	0.0 (1.8)	2.6 (1.4)	4.0 *^#^ (1.2)	1.4 (1.2)	**0.002**	**0.006**	**0.012**
Vitamin D (ug)	10 **^a^**	15 ^b^	1.4 (0.8)	2.0 (1.9)	0.5 (1.9)	2.5 ^#^ (1.8)	5.8 *^#^ (1.4)	3.2 (2.2)	**<0.001**	**<0.001**	**<0.001**
Folate (ug DFE)	250/330 **^a^**	300/400 ^b^	283 (108)	301 (101)	18 (163)	295 (110)	288 (89)	−7 (126)	0.96	0.81	0.57
Calcium (mg)	1100 **^a^**	1300 ^b^	557 (262)	510 (176)	−47 (251)	770 ^#^ (325)	1338 *^#^ (193)	568 (363)	**<0.001**	**<0.001**	**<0.001**
Iron (mg)	5.7/7.9 **^a^**	8/15 ^b^	10.4 (3.5)	11.1 (2.8)	0.7 (4.2)	12.3 (3.5)	10.0 * (2.8)	−2.3 (3.3)	0.64	0.17	**0.015**
Magnesium (mg) †	200/300 **^a^**	240/360 ^b^	160 (48)	231 (76)	71 (85)	187 (76)	227 (92)	40 (109)	0.60	**<0.001**	0.29
Phosphorus (mg)	1055 **^a^**	1250 ^b^	786 (266)	879 (194)	93 (328)	866 (328)	1483 *^#^ (230)	617 (332)	**<0.001**	**<0.001**	**<0.001**
Potassium (mg)	4500/4700 ^c^		1549 (554)	1780 (406)	231 (634)	1673 (664)	2329 *^#^ (480)	656 (658)	**0.014**	**<0.001**	**0.038**
Sodium (mg)	1500 ^c^		2863 (1281)	2521 (862)	−342 (1622)	2899 (859)	2791 (1007)	−108 (1167)	0.50	0.30	0.59
Zinc (mg) †	7.0/7.3 **^a^**	8.0/9.0 ^b^	6.1 (2.4)	6.9 (2.5)	0.8 (3.5)	7.0 (3.3)	8.7 (2.1)	1.8 (3.2)	**0.021**	**0.004**	0.33

Data are presented as mean (standard deviation). Significance set at *p* ≤ 0.05. Group (G), time (T), and interaction (Int) *p*-values are presented from the repeated-measures ANOVAs (*p* ≤ 0.05 presented in bold). Dietary reference intake (DRI) recommendations are either estimated average requirement (EAR) ^a^, recommended dietary allowance (RDA) ^b^, adequate intake (AI) ^c^, or acceptable macronutrient distribution range (AMDR) ^d^ for females aged 9–13/14–18 years old. The symbols denote when analyses were run with log-transformed data (†) and a significant difference over time within a group (*) or at a single timepoint between groups (#) based on paired-test tests or unpaired *t*-tests, respectively, conducted as post hoc testing following a significant interaction. Niacin equivalents (NE); dietary folate equivalents (DFE). Similar values for some of these variables were previously published in our other papers [20,25,31].

### 3.2. Snacking Analysis

#### 3.2.1. Snacking Characteristics (Figure 1)

**Figure 1:**
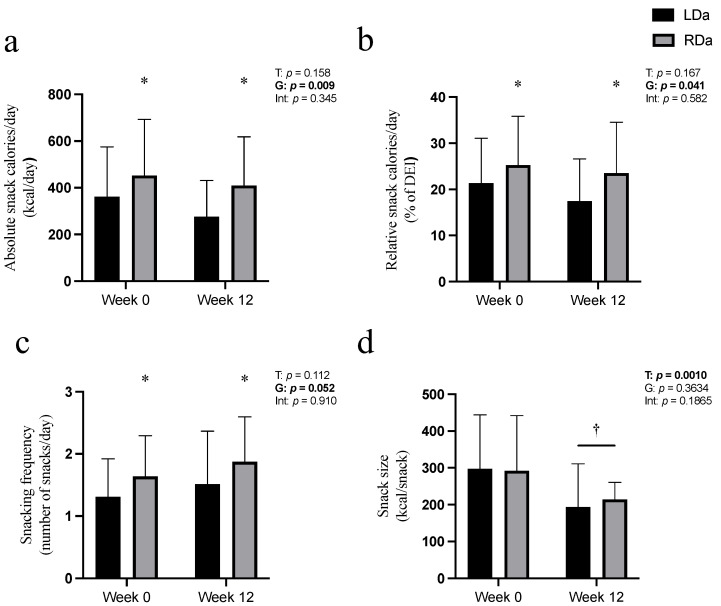
Snacking characteristics in the adolescent females with overweight/obesity in the and low dairy product intake (LDa) and recommended dairy product intake (RDa) groups before (week 0) and following the 12-week lifestyle modification intervention. Absolute snack energy intake (kcal per day) (**a**); relative snack energy intake (% of daily energy intake (DEI)) (**b**); snacking frequency (number of snacks per day) (**c**); and snack size (kcal per snack (**d**)) are displayed separately. Group (G), time (T), and interaction (Int) *p*-values are presented from the repeated-measures ANOVAs (*p* ≤ 0.05 presented in bold). Symbols denote a significant main effect of group (* *p* ≤ 0.05) and a significant main effect of time († *p* ≤ 0.05). Data are presented as mean ± standard deviation (LDa = 21, RDa *n* = 22). Absolute snack energy and snack size analyses were run with log-transformed data sets.

Absolute energy intake consumed as snacks per day (kcal/d) did not significantly change over time; however, across both timepoints, RDa consumed a greater amount of energy as snacks compared to LDa (Figure 1a). Similarly, relative energy consumed as snacks per day (% of DEI) did not change over the intervention; however, across both timepoints, RDa had a greater relative snack energy intake compared to LDa (Figure 1b). Snacking frequency (number of snacks per day) did not significantly change over time; however, across both timepoints, RDa consumed more snacks (Figure 1c). Snack size expressed as energy intake per snack (kcals/snack occasion) was lower at week 12 in both groups compared to baseline (Figure 1d). There were no significant interactions for any snacking characteristics.

#### 3.2.2. Snacking Categories (Table 5)

LDa and RDa increased their overall relative “healthy snack” consumption (and decreased their relative “unhealthy snack” consumption) during the intervention. Regarding the change over the intervention, RDa had greater increases in overall heathy snacks (and decreases in unhealthy snacks) compared to LDa. RDa had greater increases in dairy product snack consumption compared to LDa, and LDa had greater increases in vegetables and fruits snack consumption and meat and alternatives snack consumption compared to RDa. With respect to the unhealthy snack categories, RDa had greater decreases in snack drink consumption compared to LDa, whereas LDa had greater decreases in processed/mixed food consumption compared to RDa. In both these cases, the group that had greater change had greater consumption at baseline. Both groups decreased consumption of chips and cookies and desserts as snacks. There were no significant differences in candy and chocolate, condiments, or other grain-based snack consumption.

### 3.3. Dairy Product Eating Patterns Analysis (Figure 2)

**Figure 2:**
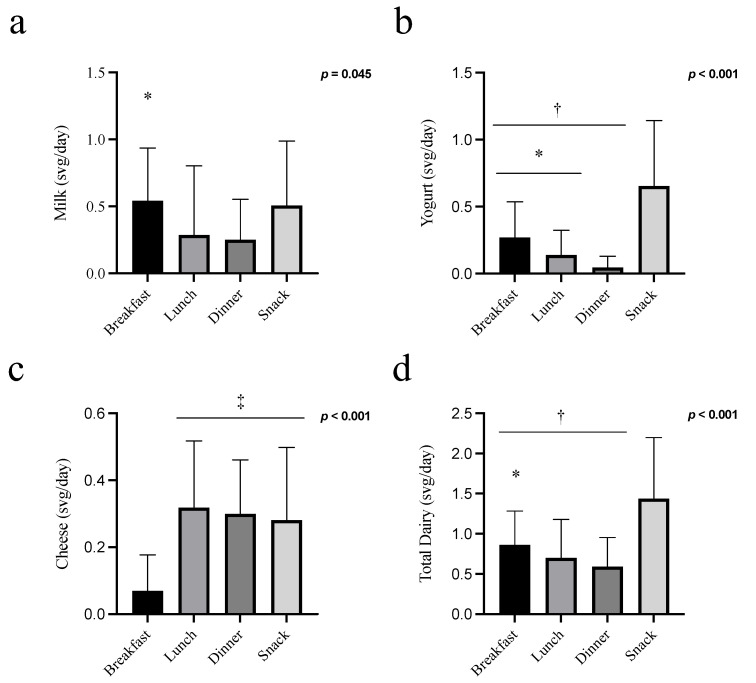
Dairy product consumption patterns in adolescent females in the recommended dairy product intake (RDa) group. Average number of svg/d of milk (**a**), yogurt (**b**), cheese (**c**), and total dairy (**d**) at different eating occasions in the week 12. Data are presented as mean ± standard deviation. F-test *p*-values are presented from the repeated measure ANOVAs (*p* ≤ 0.05 presented in bold). Symbols denote a significant difference from dinner (* *p* ≤ 0.05), snack († *p* ≤ 0.05), and breakfast (‡ *p* ≤ 0.05). Analyses were run with square-root-transformed data sets for milk, yogurt, and cheese and an outlier-treated data set for total dairy.

At week 12, among RDa participants, there was a significant effect of eating occasion for milk, yogurt, and cheese such that more servings of milk were consumed at breakfast compared to dinner (*p* = 0.013; Figure 2a), more servings of yogurt were consumed at snack, and fewer servings of yogurt were consumed at dinner compared to all other eating occasions (*p* < 0.05, Figure 2b), and fewer servings of cheese were consumed at breakfast compared to all other eating occasions (*p* < 0.001, Figure 2c). Overall, participants consumed a greater number of servings of “total dairy” at snack compared to all other mealtimes (breakfast *p* = 0.006; lunch *p* = 0.002; dinner *p* = 0.001; Figure 2d) and at breakfast compared to dinner (*p* = 0.015). “Other dairy” products (including ice cream, cream cheese, and sour cream) contributed only 1.7 (3.0) % of total energy intake at week 12 and were not explored further.

## 4. Discussion

Our results demonstrate that increased dairy product consumption, *via* provision of dairy products, during a lifestyle modification, weight management intervention that included diet and exercise, improved nutrient intake and healthy snack consumption in adolescent females with OW/OB to a greater extent than the same intervention in the group with habitually low dairy product consumption. Increases in overall diet quality, assessed by total HEI-C score, and decreases in snack size (kcal/snack) in both groups were also observed. In addition, more servings of dairy products were consumed as snacks than at other meal occasions by the RDa participants. Overall, these results suggest that individual, RD-directed, healthy eating dietary counselling during the lifestyle modification intervention improved diet quality and nutrient intakes and that the consumption of dairy products, in accordance with participants’ personal choices, improved the intake of several key nutrients and snacking characteristics.

### 4.1. Diet Quality and Nutrient Intake

Several cross-sectional analyses have demonstrated favourable associations between dairy product intake and indices of a healthy diet, including an increased intake of key nutrients (e.g., calcium and vitamin D) and improved diet quality scores (e.g., Healthy Eating Index 2015), in children and adolescents [5,6,32,33,34]. However, RCT data assessing the effect of increased dairy product consumption on diet quality scores (i.e., HEI-C) in adolescents are scarce. Our RCT provides novel data on the effect of increased dairy product intake on diet quality, assessed using HEI-C, in female adolescents with OW/OB. Specifically, larger improvements in the “milk and alternatives” (by design) and “other” food sub-scores in RDa were found compared with LDa. The latter indicated that there was a larger reduction in the relative energy contribution of the “other” category, which is comprised of foods with no serving size recommendations in the previous CFG (e.g., confectionary, SSBs). The reduction in “other food” sub-score is notable, as several studies have reported that increased consumption of SSBs and ultra-processed foods, which are categorized as “other food”, are associated with adverse health outcomes [35,36,37,38]. Results also demonstrated greater improvements in the RDa group for several key nutrients preferentially found in dairy products, including vitamin D, calcium, phosphorus, and potassium, the intakes of which are low in adolescents [39]. The improved nutrient intakes in the RDa group align with a recent observational study using Canadian Community Health Survey data demonstrating that “milk and alternatives” products are major sources of calcium, vitamin D, and vitamin B12 for Canadian adolescents [9].

The RDa participants consumed mixed dairy products, ranging in fat content, to meet the recommended daily servings [26]. Dairy products have been identified as contributors to overall saturated fat intake in several studies in youth [9,40,41,42]. However, in our study, despite increased dairy product consumption (including full-fat cheese), we observed no change in the “saturated fat” HEI-C sub-score in the RDa group, suggesting that other sources of saturated fat (e.g., from the meat and alternatives or “other food” categories) were reduced. There is controversy regarding whether dietary guidelines should blanketly recommend lower saturated fat intakes [43,44], and there is evidence that the association between saturated fat and disease risk is influenced by food source [45,46]. A recent review [47] summarized evidence from several systematic reviews and meta-analyses demonstrating inverse or neutral associations between the consumption of full-fat dairy products and adverse cardiometabolic health outcomes. The HEI-C is a diet quality tool that combines recommendations on several components of nutrition and is impacted by both wholefoods and single nutrients (particularly the nutrients of public health concern). In relation to (high-fat) dairy products, while evidence demonstrates that saturated fat within the dairy wholefood matrix is purportedly not associated with increased disease risk [45,47], within the HEI-C scoring paradigm, dairy product consumption improves the adequacy component sub-score of “milk and alternatives” yet worsens the “saturated fat” moderation component sub-score. Given that we observed divergent effects of increased dairy product consumption on wholefood and single-nutrient components of the HEI-C and that it can be challenging to consider and reconcile these different aspects of diet quality concurrently [48], it may be informative for future lifestyle intervention studies aiming to increase dairy product consumption and examine diet quality to consider the contribution of different food sources to saturated fat intake and to focus on diet-quality scoring systems or analysis methods based on the relative contribution of nutrients from wholefoods vs. processed foods and different dietary patterns. Nonetheless, while we demonstrated similar overall changes in HEI-C scores between RDa and LDa groups, we also demonstrated more favourable calcium, vitamin D, phosphorus, and potassium intakes in RDa. In contrast, iron intake decreased to a greater extent in RDa compared to LDa over the intervention. “Milk and alternatives” foods contribute a limited amount of daily iron intake in adolescents [9]. While increased consumption of dairy products may have replaced foods containing iron in the RDa group, the mean iron intake at the end of the intervention was not different between groups and was still above the EARs for females aged 9–13 and 14–18 years in both groups. Similarly, sugar intake decreased to a greater extent in LDa compared to RDa, which is consistent with a recent investigation demonstrating that dairy products contribute to increased total sugar intake in Canadian adolescents [49]. Importantly, dairy products are nutrient-dense contributors to total sugar intake in Canadian adolescents as opposed to confectionary and regular soft drinks (i.e., SSBs), and adolescents with mean intakes of total sugar had higher intakes of several nutrients important for growth and development that are found in dairy products, specifically potassium and calcium, compared to those with lower intakes of total sugar [49].

### 4.2. Snacking Characteristics and Categories

Assessing snack food preference in adolescents with OW/OB is important given that snacking on healthy foods such as vegetables, fruits, and protein-containing foods such as dairy products may aid in promoting satiety [15] and reaching nutrient requirements [15,16]. However, intervention studies assessing snack consumption have only examined single snack food items (i.e., specific salty or sugary foods) consumed at any eating occasion [50,51,52,53,54] and have not conducted a comprehensive assessment of snacking behaviours. Nonetheless, nutrition and/or physical activity education interventions have succeeded in decreasing the consumption of unhealthy snack food items, including SSBs [50,55], desserts [53], and fast food [51] in adolescents. Future research should examine additional snacking details including snacking time, situation, location, reasons, etc., and involve adolescents in the development of healthy snacking guidelines [56], as these details represent other degrees of freedom to manipulate to achieve more healthful snacking overall [13].

Dairy products were chosen as snacks among the RDa participants and successfully replaced unhealthy snack consumption, as shown by the decrease in the relative contribution of chips/cookies, desserts, processed foods, and drinks to snack consumption. Prior to the intervention, participants consumed flavoured coffee beverages, including iced cappuccinos and “frappuccinos”, which are classified as caffeinated SSBs. Similarly, previous research has also indicated an increased consumption of these energy-dense, nutrient-poor, flavoured coffee drinks, which are high in sugar and caffeine [57]. In the RDa group, participants may have replaced these drink choices with that of white or chocolate milk as a snack. Indeed, previous research has shown that female adolescents who consume milk (white or chocolate) tend to consume fewer SSBs than those who do not drink milk [58]. Healthy snack consumption also improved among the LDa group (although to a lesser extent), indicating that our nutritional counselling overall was efficacious in both groups for improving snack choice in adolescents with OW/OB.

### 4.3. Dairy Product Eating Patterns

Our study also examined dairy product eating patterns. Cross-sectional studies in Australia have demonstrated that the highest proportion of individuals consume dairy products [59] or milk [60] at breakfast, followed by snack occasions. Similarly, a recent Canadian study found that a higher proportion of dairy servings were consumed at breakfast, followed by snack occasions [14]. Although the RDa participants in our study consumed more servings of dairy products as snacks rather than at breakfast, this difference may relate to the participants consuming a greater number of dairy servings overall compared to the majority of Canadian adolescents [9]. Additionally, consumption preferences differed by dairy product. For example, yogurt was preferred at snack vs. all other meal occasions, whereas cheese was consumed least often at breakfast, and milk was preferred at breakfast over dinner. As participants were not instructed on how or when to consume these foods, aside from the one serving of milk post exercise (which was removed from the snacking- and patterns-based analyses), the design of our RCT characterizes the self-selected consumption of the various dairy products. Thus, our study offers valuable information and insight on dairy product preferences and eating patterns that should be used in future individual counselling and public health efforts to help increase intakes in this demographic. For example, when encouraging consumption of yogurt, it may be prudent to target snack occasions. Moreover, our RCT data are of particular interest because adolescent females pose unique challenges related to dairy product intake given that their consumption of dairy products has consistently been low [9], and they have been shown to actively avoid dairy products for various reasons including its higher fat content and its perceived association with other adverse outcomes (e.g., weight gain) [61]. Given the former point, providing a variety of dairy products (milk, yogurt, and cheese) with the ability for autonomous consumption may be a good strategy, as this produced a high level of compliance with dairy product intake reported in [20], and anecdotally, consumption was very well-tolerated.

### 4.4. Limitations

Our study had some limitations. The dietary analyses in this study relied on self-reported food records. While food records are commonly used to measure dietary intake in nutrition research, there is evidence of inaccurate self-reporting of food intake [62]. Indeed, female adolescents with OW/OB have been shown to underreport dietary intake [63], and underreporting is positively associated with OW/OB in adolescent cohorts [64,65]. However, food records and self-reported dietary intake remain a common practice in the field due to feasibility and the lack of available and validated biomarkers to reflect accurate dietary intake [62]. To mitigate the issue of inaccurate reporting in our study, participants met and reviewed food records in detail with the RD throughout the intervention. Second, our sample size was smaller, and findings may not be generalizable to other populations, including male adolescents and younger children with OW/OB. Lastly, our results are limited to Canadian food products and North American eating patterns. Differences in dietary guidelines, fortification practices, eating patterns, and sociocultural factors across geographic locations may impact the influence of dairy product consumption on the dietary outcomes reported herein.

## 5. Conclusions

This study demonstrated that consuming a variety of dairy products (4 svg/d) during a lifestyle modification, weight management intervention improved nutrient intake and total relative healthy snack consumption compared to a low-dairy diet (≤2 svg/d) in adolescent females with OW/OB. Both groups improved overall diet quality, intake of some nutrients, and decreased snack size following the 12-week intervention, providing additional support for the efficacy of our lifestyle modification intervention involving individual nutritional counselling on these outcomes. Given these and our previous findings [20,25], it may be beneficial to promote dairy product consumption in this population to provide crucial nutrients to support overall health during this critical time of musculoskeletal growth and development. Future studies with a greater sample size and in other populations should be done to investigate the influence of increased dairy product consumption on diet quality, nutrient intakes, and snacking patterns to assess more generalizable effects.

## Figures and Tables

**Table 1:** Descriptive data for the female adolescent participants in the low dairy product intake (LDa; *n* = 23) and recommended dairy product intake (RDa; *n* = 24) groups.

	LDa	RDa
Age (years)	14.9 (2.3)	14.7 (2.2)
Height (cm)	163.6 (5.9)	163.6 (7.9)
Weight (kg)	79.2 (13.4)	80.8 (15.3)
BMI (kg/m^2^)	29.6 (5.0)	30.2 (5.1)

Data are expressed as mean (standard deviation). Body mass index (BMI).

**Table 2:** Examples of the most common food items in the eleven distinct categories of snacks.

Category	Examples
Healthy Snacks	
1. Dairy products	Cheese, milk, yogurt
2. Grains (breads and cereals)	Bread, bagels, pasta, ready to eat cereals, waffles
3. Meat and alternatives	Chicken, egg, hummus, nuts
4. Vegetables and fruits	Berries, apples, carrots, cucumber
Unhealthy Snacks	
5. Candy and chocolate	Chocolate bars, gummy candies, hard candies
6. Chips and cookies	Potato chips, tortilla chips, chocolate chip cookies
7. Condiments	Butter, salad dressing, maple syrup, jam
8. Other grain-based snacks	Crackers, rice cakes, popcorn, granola bars, pretzels, energy bars
9. Desserts	Brownies, cake, frozen desserts (ice cream), muffins
10. Drinks	Sweetened tea, coffee beverages, juice
11. Processed/mixed foods	Pre-made sandwiches and smoothies, fast-food

**Table 5:** Snacking patterns in adolescent females with overweight/obesity in the low dairy product intake (LDa) and recommended dairy product intake (RDa) groups at weeks 0 and 12 of the intervention.

	LDa (*n* = 21)			RDa (*n* = 22)			*p*-Values	
Snacking Categories (% of Snack Kcal)	Wk 0	Wk 12	Change	Wk 0	Wk 12	Change	Time	Change
**Healthy**	22 (23)	53 (28)	31 (27)	33 (20)	82 (17)	50 (28)	**<0.001**	**0.036**
Dairy products	4.8 (6.3)	1.4 (4.0)	−3.4 (8.2)	8.8 (12)	64 (25)	55 (32)	**<0.001**	**<0.001**
Grains	4.0 (6.4)	1.4 (3.9)	−2.6 (7.5)	8.7 (11)	3.0 (6.7)	−5.8 (11)	**0.003**	0.37
Meat and alternatives	1.3 (2.1)	23 (29)	22 (29)	2.2 (6.7)	4.3 (10)	2.1 (13)	**<0.001**	**0.025**
Vegetables and fruits	12 (23)	27 (21)	15 (30)	13 (20)	11 (12)	−1.6 (23)	**0.010**	**0.008**
Unhealthy *	78 (23)	47 (28)	−31 (27)	67 (20)	18 (17)	−50 (28)	**<0.001**	**0.036**
Candy and chocolate	5.9 (13)	1.7 (3.7)	−4.2 (13)	6.1 (11)	3.0 (6.2)	−3.1 (14)	0.14	0.99
Chips and cookies	16 (17)	11 (16)	−5.6 (21)	13 (14)	1.6 (4.0)	−11 (14)	**0.001**	0.43
Condiments	3.2 (5.0)	2.6 (4.3)	−0.7 (6.7)	2.0 (3.1)	1.2 (3.3)	−0.8 (2.7)	0.46	0.14
Desserts	13 (14)	12 (22)	−0.5 (29)	13 (13)	3.4 (7.0)	−9.9 (14)	**0.006**	0.46
Drinks	7.4 (11)	4.0 (9.1)	−3.4 (16)	17 (17)	2.2 (3.9)	−15 (17)	**<0.001**	**0.032**
Other grain-based snacks	17 (24)	13 (17)	−4.0 (23)	11 (11)	4.1 (6.0)	−6.9 (13)	0.13	0.077
Processed/mixed foods	16 (20)	2.5 (8.6)	−13 (21)	5.6 (11)	2.3 (4.2)	−3.3 (10)	**0.004**	**0.031**

Data are presented as mean (standard deviation). Significance set at *p* ≤ 0.05. The *p*-values are from the Wilcoxon matched pairs signed-rank test to compare week 0 and 12 values and the Mann–Whitney U test to compare changes over the intervention between groups are shown (*p* ≤ 0.05 presented in bold). * “Unhealthy” snack consumption (percent of total snack kcal) represents the inverse of “healthy” snack consumption.

## Data Availability

The datasets used and/or analysed during the current study are available from the corresponding author on reasonable request.

## Supplementary Materials

The following supporting information can be downloaded at: https://www.mdpi.com/article/10.3390/children9111703/s1, Figure S1: CONSORT Flow diagram; Table S1: CONSORT checklist.
