# Supplementary material for: The Influence of Increased Dairy Product Consumption, as Part of a Lifestyle Modification Intervention, on Diet Quality and Eating Patterns in Female Adolescents with Overweight/Obesity
Source: Children (Basel). 2022 Nov 6;9(11):1703. doi: 10.3390/children9111703 (PMC9688836; doi:10.3390/children9111703)
Supplement: Supplementary file 1 [file children-09-01703-s001.zip › children-1974662-supplementary/Consort Diagram - Supplementary Figure S1 .pdf]

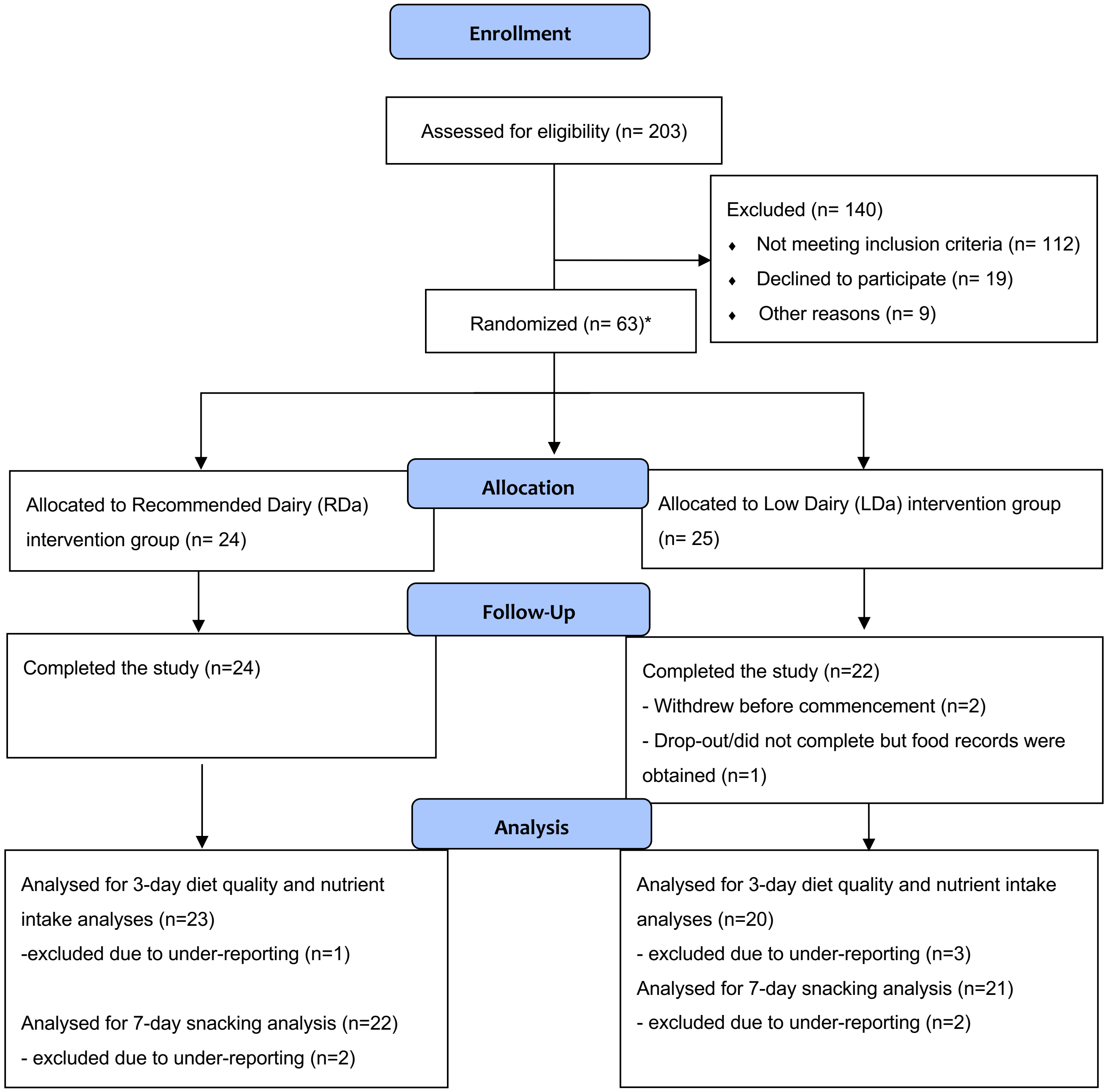

Supplementary Figure S1: Consort Flow Diagram.

*The remaining participants were allocated to a no-intervention control group not reported in this study.
